# Supplementary material for: Exploring the Role of Hypoxia-Inducible Carbonic Anhydrase IX (CAIX) in Circulating Tumor Cells (CTCs) of Breast Cancer
Source: Biomedicines. 2023 Mar 17;11(3):934. doi: 10.3390/biomedicines11030934 (PMC10046014; doi:10.3390/biomedicines11030934)
Supplement: Supplementary file 1 [file biomedicines-11-00934-s001.zip › biomedicines-2244459-supplementary.pdf]

Table S1: indicating the subtyping per ATCC, disease classification per ATCC, ability to form spheres under suspension culture, and anoikis resistance or sensitivity through seven days of suspension culture.

Cell Line	Subtyping*	Tissue	Disease Classification	Sphere Forming (Y/N)	Anoikis Resistant/ Sensitive
HCC1937	TNBC (Basal-Like)	Breast; Duct; Mammary gland	Primary ductal carcinoma; Stage IIB, grade 3;	N	Sensitive
BT20	TNBC (Basal-Like)	Breast; Mammary gland	Breast tumor carcinoma	Y	Resistant
HCC38	TNBC (Claudin-Low)	Breast; Duct; Mammary gland	Stage IIB, Grade 3, with 3/28 lymph node metastasis.	N	Sensitive
MB-231	TNBC (Claudin-Low)	Breast; Mammary gland	Adenocarcinoma	N	Resistant
HS578T	TNBC (Claudin-Low)	Breast; Mammary gland	Carcinoma	N	Sensitive
BT549	TNBC (Claudin-Low)	Breast; Mammary gland	Papillary, invasive ductal carcinoma with metastasis to 3 of 7 regional lymph nodes	Y	Resistant
T47D	ER^+/ PR^+	Breast; Mammary gland	Infiltrating ductal carcinoma with pleural effusion	Y	Resistant
MCF7	ER^+	Breast; Mammary gland	Breast adenocarcinoma with pleural effusion	Y	Resistant
ZR75-1	ER^+	Breast; Duct; Mammary gland	Ductal carcinoma	N	Resistant
BT474	ER^+/ PR^+/ $HER2^+$	Breast; Duct; Mammary gland	Solid, invasive ductal carcinoma	Y	Resistant
AU565	$HER2^+$	Breast; Mammary gland	Breast adenocarcinoma with pleural effusion	N	Resistant
SKBR3	$HER2^+$	Breast; Mammary gland	Breast adenocarcinoma with pleural effusion	N	Resistant

*Triple negative breast cancer, TNBC; Estrogen receptor positive, ER^+; Progesterone receptor positive, PR^+; Human epidermal growth factor receptor 2 positive, $HER2^+$; ATCC, American Type Culture Collection (http://www.atcc.org/).

Table S2. Signaling pathway analysis performed using DAVID on the genes that were significantly upregulated or downregulated in both MB-231 and MCF7 cell lines. The significant GO_BP Terms with the associated p-values and genes are shown (False Discovery Rate (FDR) <0.05, p<0.05).

	**Gene Ontology Term: Biological Process**		**P-Value**	**Gene Count**	**Genes**
**Upregulated**	GO:0010628	Positive regulation of gene expression	0.0004	6	GJA1, NFIL3, FN1, ACTG2, HSPA1B, VEGFA
	GO:0001666	Response to hypoxia	0.0079	4	CA9, ADM, ANGPTL4, VEGFA
	GO:0030198	Extracellular matrix organization	0.0113	4	CSGALNACT1, ELF3, LOX, FN1
	GO:0061621	Canonical glycolysis	0.0017	3	PFKFB4, ENO2, HK2
	GO:0002576	Platelet degranulation	0.0250	3	FN1, TUBA4A, VEGFA
	GO:0045766	Positive regulation of angiogenesis	0.0306	3	ADM, ANGPTL4, VEGFA
**Downregulated**	GO:0045087	Innate immune response	0.0202	4	APP, MAVS, TRIM25, C4BPB
	GO:0032480	Negative regulation of type I interferon production	0.0403	2	MAVS, TRIM25

Table S3. The top KEGG_PATHWAY from the DAVID analysis for the upregulated and downregulated gene panels. (False Discovery Rate (FDR) <0.05, p<0.05)

	**KEGG_PATHWAY**		**P-Value**	**Gene Count**	**Genes**
**Upregulated**	hsa04066	HIF-1 signaling pathway	0.0038	4	SLC2A1, ENO2, HK2, VEGFA
**Downregulated**	hsa04622	RIG-I-like receptor signaling pathway	0.0074	3	MAVS, RNF125, TRIM25

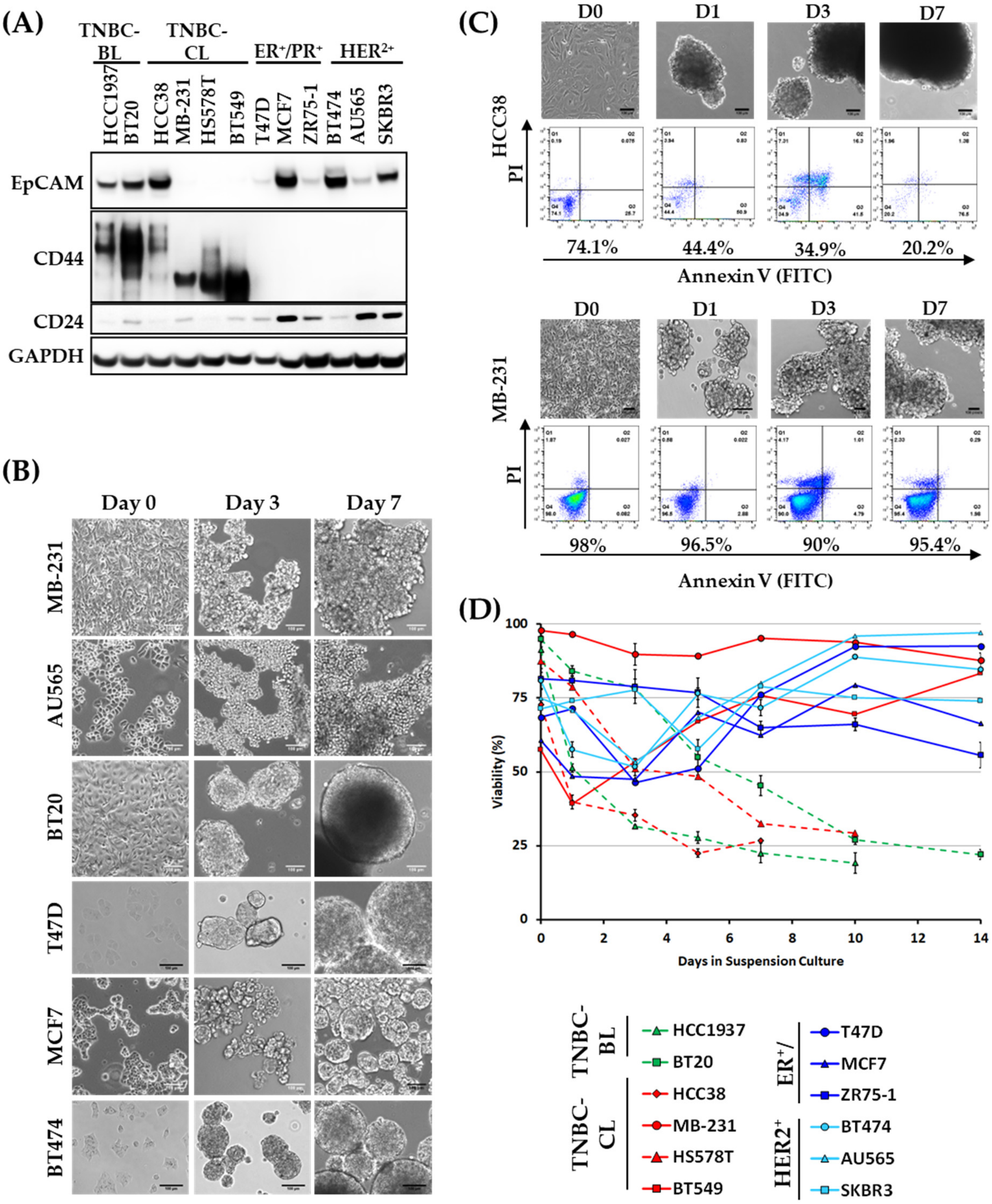

Figure S1. Immunoblot analysis of panel of monolayer cultured BCC lines for CSC markers and cell line screening for anoikis resistance over 14 days of suspension culture. (**A**) A panel of monolayer cultured breast cancer cell lines were screened for the stem cell markers of CD44 and CD24 as well as the epithelial marker of EpCAM using immunoblotting. (**B**) Brightfield microscopy was performed at days 0, 3, and 7 of suspension culture to determine whether cell lines formed spheroids under suspension culture. Scale bar = 100 μm. (**C**) The panel was screened for anoikis resistance following monolayer culture (D0) and at the indicated timepoints over 14 days of suspension culture using bright field imaging and a flow

cytometry apoptosis assay. (**D**) Viability (no detection of anti-annexin V-FITC or propidium iodide) was assessed over 14 days of culture. Cell lines were determined anoikis resistant if viability was maintained over 50% during suspension subculture. Triple negative breast cancer- basal-like, TNBC-BL; Triple negative breast cancer- claudin low, TNBC-CL

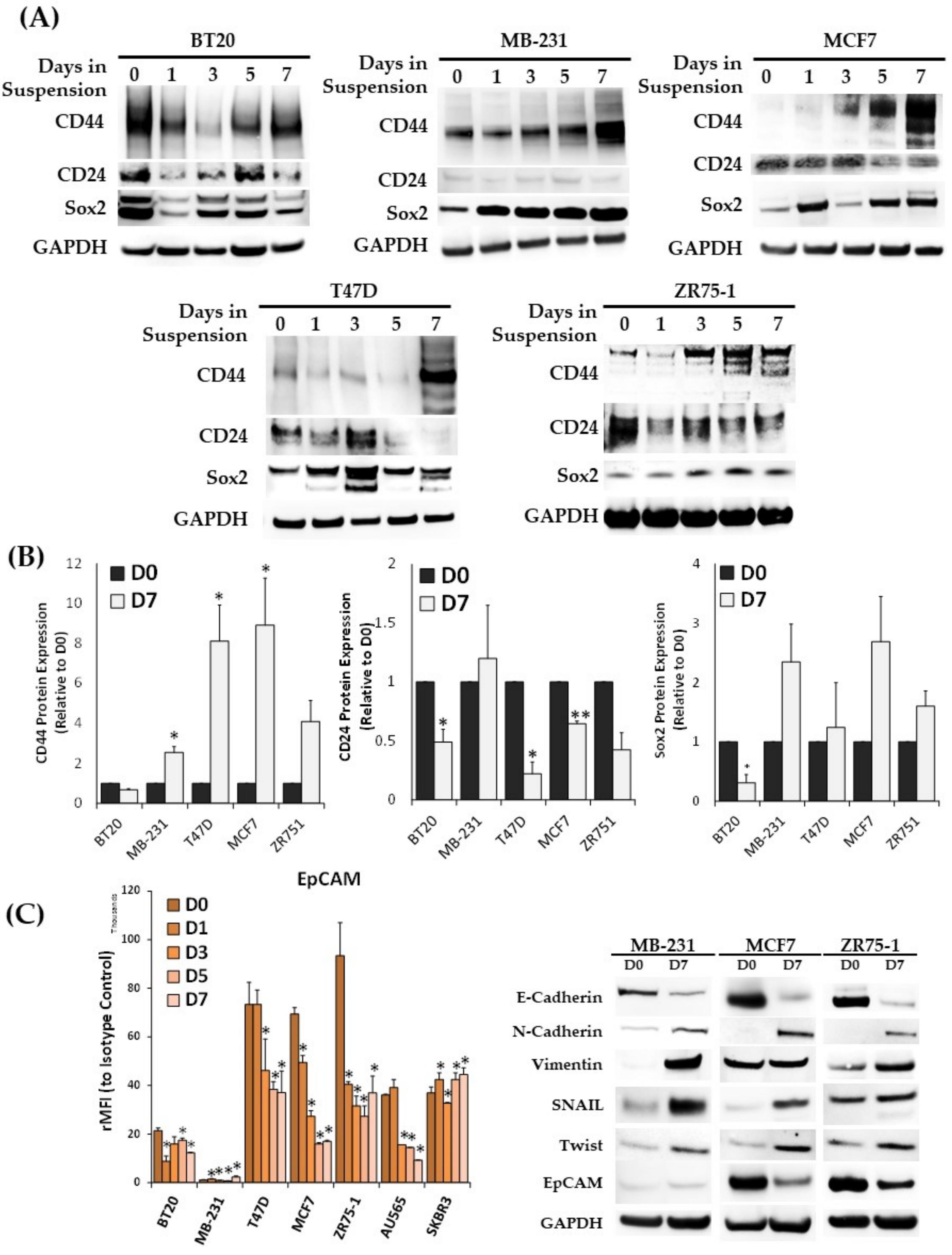

Figure S2. Immunoblot and flow cytometry analysis of BCCs cultured in suspension for CSC and EMT markers. (**A**) The panel of BCC lines was analyzed for CD44, CD24, and Sox2 expression at the indicated time points following suspension condition or monolayer culture (D0). Immunoblot images are representative of 3 biological replicates. (**B**) Relative protein expression is shown to monolayer (day 0) (relative densitometric analysis). (N≥3; *p<0.05 to D0). (**C**) EMT markers were analyzed in the panel of BCCs cultured in suspension over the 7 days. EpCAM surface expression (relative median fluorescence to isotype control) was shown to decrease over the 7 days of culture relative to monolayer. (N=3; *p<0.05 to D0). EMT protein expression was analyzed using immunoblotting of MB-231, MCF7, and ZR75-1 cells cultured for 0 or 7 days in suspension. All statistics were performed with Graph Pad Prism, with an unpaired t-test with Welch's Correction.

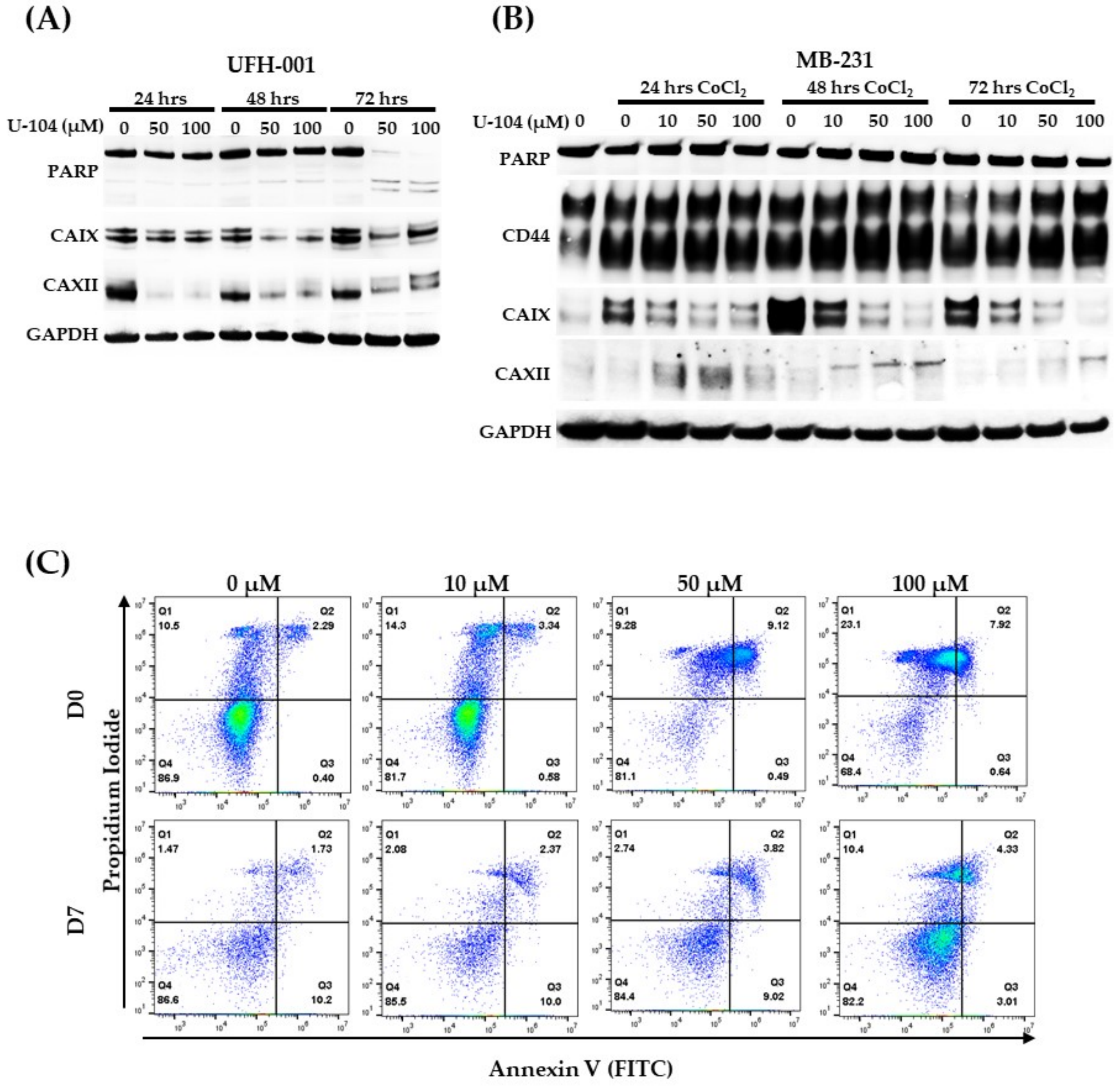

Figure S3. Determination of the impact of a low, medium, and high concentration of U-104 on viability and hypoxia-related protein expression on UFH-001 and MB-231 cells. (**A**) UFH-001 cell line was

analyzed following U-104 treatment for 24, 48, or 72 hours in monolayer culture. By 72 hours, treatment with U-104 resulted in cell death, as visualized by cells lifting off of monolayer and PARP cleavage and degradation. 50 μM of treatment was sufficient to reduce CAIX and CAXII expression. (**B**) MB-231 cells were treated with 200 μM of $CoCl_2$ for a chemical simulation of hypoxia for 24, 48 or 72 hours containing 0, 10, 50, or 100 μM of U-104 at which point cells were harvested for immunoblotting. CD44 total expression was not impacted by the U-104 treatment when stimulated by $CoCl_2$. $CoCl_2$ treatment stimulated CAIX expression, which was reduced when treated with U-104 in a dose-dependent manner. Viability as reported through PARP cleavage was not impacted. (**C**) MB-231 cells were cultured in monolayer (D0) or suspension culture for 7 days (D7) and then treated with U-104 for 24 hours. Viability was assessed using an apoptosis flow cytometry assay.
